# Transcutaneous Ablation of Lung Tissue in a Porcine Model Using Magnetic-Resonance-Guided Focused Ultrasound (MRgFUS)

**DOI:** 10.3390/tomography10040042

**Published:** 2024-04-06

**Authors:** Jack B. Yang, Lauren Powlovich, David Moore, Linda Martin, Braden Miller, Jill Nehrbas, Anant R. Tewari, Jaime Mata

**Affiliations:** 1 Department of Radiology & Medical Imaging, University of Virginia, Charlottesville, VA 22903, USA; jrv4sg@virginia.edu (J.B.Y.); bmm3ab@virginia.edu (B.M.);; 2 Focused Ultrasound Foundation, Charlottesville, VA 22903, USA; 3 Section of Thoracic Surgery, Department of Surgery, University of Virginia, Charlottesville, VA 22903, USA; lm6yb@uvahealth.org

**Keywords:** magnetic-resonance-guided focused ultrasound surgery, lung ablation, lung cancer

## Abstract

Focused ultrasound (FUS) is a minimally invasive treatment that utilizes high-energy ultrasound waves to thermally ablate tissue. Magnetic resonance imaging (MRI) guidance may be combined with FUS (MRgFUS) to increase its accuracy and has been proposed for lung tumor ablation/debulking. However, the lungs are predominantly filled with air, which attenuates the strength of the FUS beam. This investigation aimed to test the feasibility of a new approach using an intentional lung collapse to reduce the amount of air inside the lung and a controlled hydrothorax to create an acoustic window for transcutaneous MRgFUS lung ablation. Eleven pigs had one lung mechanically ventilated while the other lung underwent a controlled collapse and subsequent hydrothorax of that hemisphere. The MRgFUS lung ablations were then conducted via the intercostal space. All the animals recovered well and remained healthy in the week following the FUS treatment. The location and size of the ablations were confirmed one week post-treatment via MRI, necropsy, and histological analysis. The animals had almost no side effects and the skin burns were completely eliminated after the first two animal studies, following technique refinement. This study introduces a novel methodology of MRgFUS that can be used to treat deep lung parenchyma in a safe and viable manner.

## 1. Introduction

Lung cancer is the second most diagnosed cancer amongst both men and women in the United States and is the leading cause of cancer-related death for both genders, with an estimated 127,070 deaths in 2023 [1]. For early-stage non-small cell lung cancer (NSCLC), the mainstay treatment is anatomic surgical resection [2]. However, even following surgical treatment, the five-year survival rate for those treated with surgical interventions is only 56–90%, compared to a survival rate of 6% for those who remained untreated [3]. Up to 20% of patients with stage I/II NSCLC are unsuitable for surgery, and those who are surgically treated face potential complications, with a 0.3–2% incidence of operative mortality [4,5]. As such, it has become increasingly important to discover non-invasive, accurate, and better alternative treatments for NSCLC.

Magnetic-resonance-guided focused ultrasound (MRgFUS) is a treatment modality that is non-invasive and has been used in clinical practice for the treatment of uterine fibroids, prostate cancer, and prostate hyperplasia, as well as for the treatment of essential tremor, among other diseases [6,7]. Using high-energy ultrasound waves, acoustic energy can be concentrated to heat a deep tissue target. If live tissue is heated to more than 57–60 degrees Celsius for a few seconds, coagulative necrosis occurs and causes rapid cell death. MRgFUS is highly accurate as the high anatomical visualization from magnetic resonance imaging (MRI) allows for the controlled targeting of the focused ultrasound (FUS) beam, as well as the real-time observation of ablated tissues. MRI-guided technology can also be used to map thermal energy and provide heat quantification, allowing for an increased accuracy and decreased damage to collateral tissue [8]. As a result, MRgFUS is a progressive technique for the surgical removal of benign or malignant masses.

One of the issues with the use of MRgFUS for the treatment of lung cancer is the air within the lung, which attenuates ultrasound frequency waves, even at low levels of lung inflation [9]. Some studies have utilized the one-lung flooding (OLF) technique, in which one of the lungs is filled with saline while the other remains ventilated [10]. However, this technique brings the risk of post-flooding complications, such as the introduction of extracorporeal organisms into the lung parenchyma, respiratory compromise, as well as the incomplete removal of the liquid medium, which can be a good ground for bacterial growth. Furthermore, histologically, there is an increase in inflammatory cells following OLF [11], and it is unknown what its long-term effects are on survival.

A less invasive alternative for increasing the conductive accuracy and strength of MRgFUS beams is the use of a controlled hydrothorax. When the pleural space is flooded, this creates an acoustic window for MRgFUS, which could then be used to ablate deep tissue within the thoracic cavity. This avoids the possibility of any complications associated with pulmonary edema, and most fluid within the pleural space could be reliably removed using thoracentesis. In addition, as the re-absorption rate of the pleural space increases in response to excess fluid, the remaining fluid not removed using thoracentesis would be re-absorbed [12]. An in vivo porcine model with artificial pleural effusion combined with MRgFUS showed success in reaching the required temperature for the thermal ablation of tissue [13]. When this technique was used to treat 49 patients with hepatocellular carcinoma, there was an effectiveness rate of 79.5%, thus indicating that FUS waves conducted through pleural effusion are clinically viable for deep tissue ablation [14].

It has not been previously studied whether a controlled hydrothorax can be used to treat the deep parenchyma of the lung with MRgFUS. If a hydrothorax is combined with intentional lung collapse, there are reduced air–tissue interfaces, thus allowing for increased consistency in the targeting of lung tissue and strength of MRgFUS beams. Accordingly, this project proposes and tests the feasibility of a minimally invasive procedure using a controlled hydrothorax and intentional lung collapse for the transcutaneous ablation of lung tissue using MRgFUS.

## 2. Materials and Methods

The research protocol was approved by our Institutional Animal Care and Use Committee (IACUC). For this study, a total of eleven pigs were treated with MRgFUS ablation (two Yucatán mini and nine Yorkshire). The two Yucatán mini pigs were sourced from Sinclair Bio Resources and the nine Yorkshire pigs were obtained from Mary B Farms. 

Pigs were treated with subcutaneous MRgFUS using an Exablate 2000OR system (Insightec, Tirat Carmel, Israel), with a portable patient table docked to a 3 T MR scanner (General Electric, Chicago, IL, USA). The system is capable of ablating tissue with a volume of 12 mm × 12 mm × 30 mm and utilizes a focused piezoelectric transducer array with 120 mm diameter [15]. The transducer is a spherically curved concentric ring with 8 sectors and 208 independently phased elements, which can be independently controlled [16]. The transducer can be mechanically controlled to change the position and angle, while depth and the focal distance are controlled electronically through Insightec Exablate 2000 software. Each of these is visible on the Exablate 2000 targeting software and was adjusted manually to avoid as much interference as possible by the ribs (i.e., by shifting the transducer to gaps between the ribs). The embedded thermometry in the Insightec system was used to estimate the temperature of targeted tissue during ablation.

**Figure 1:**
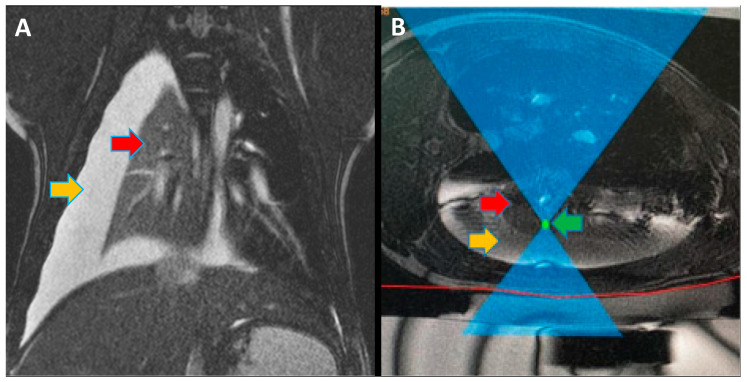
(**A**) Magnetic resonance imaging (MRI) coronal view of pig #10 during magnetic-resonance-guided focused ultrasound (MRgFUS) treatment. Red arrow shows the collapsed non-ventilated right lung. Orange arrow shows the thoracic cavity filled with saline to create an acoustic window. (**B**) MRI axial view of pig #8 during MRgFUS treatment planning. Red arrow shows a collapsed non-ventilated left lung. Orange arrow shows the thoracic cavity filled with saline to create an acoustic window. Green arrow shows the focused ultrasound (FUS) focal spot targeting one of the ablation areas.

Before the procedure, Insightec Exablate software was used for the 3-dimensional planning of the treatment (Figure 1). For pigs #1–4, 45 mm UF gel pads (Insightec, Tirat Carmel, Israel) were used for cutaneous cooling, while for pigs #5–11, 25 mm UF gel pads were used (Insightec, Tirat Carmel, Israel). 

### 2.1. MRgFUS Ablations

To prepare the pigs for MRgFUS lung ablation, atropine (4 mg/kg) was administered to decrease airway secretions for easier bronchoscopy visualization. Following this, the pigs were anesthetized (induction with 6 mg/kg Telazol and 2 mg/kg Xylazine, maintenance with 2% Isoflurane) and monitored for hemodynamic stability with pulse oximetry, electrocardiography, and blood pressure tests. One of the main bronchi was then intubated with a double-lumen endotracheal tube, ranging from sizes 28 to 35 French, depending on the size of the pig. Intubation was confirmed with a pediatric bronchoscope, and one lung was mechanically ventilated with a tidal volume of 200 to 300 cc while the other lung was allowed to collapse. All pigs except for Pig #10 had the left lung collapsed, since it was the target lung for the MRgFUS, while the right lung was artificially ventilated. Pig #10 had the left lung ventilated and the right lung collapsed due to difficulties in endotracheal intubation. A 2 cm incision was then made in the thorax in the space between the 4th and 5th ribs, and lung collapse was confirmed through a video thoracoscope. Following confirmation, 500 to 1000 cc of warm saline was introduced into the hemithorax through the incision. Fiberoptic bronchoscope was used to visualize the pleural cavity until approximately 90% of the cavity was filled.

For pigs #10 and #11, a new methodology was introduced to create a simulated MRI target to ablate. A combination of Vitamin E (MRI marker), Evans blue (histology marker), and green food dye (visual marker) was mixed and then injected (0.25–0.5 mL) into the parenchyma of the lung (2–10 mm depth), using a spinal tap needle. This was observed through video thoracoscopy to ensure the right location and that the mixture was properly injected into the parenchyma and not into the pleural space.

Following the controlled hydrothorax, animals were transported to the MRgFUS suite, and placed in a lateral position on the MRgFUS table with the collapsed lung as the dependent lung. While in the MRI scanner, a ventilator-induced breath-hold was conducted during maximum inspiration of the ventilated lung, and ablations were conducted. An example of the software and planning used for MRgFUS ablations is shown in Figure 1. The first five pigs were treated through an escalation procedure to best determine the power required for successful ablation with minimal side effects (i.e., skin burns). The ablation energies and number of focal spots are described in Table 1.

For pigs #5–11, the gel pad between the table and skin was switched from a 45 mm thick to a 25 mm thick gel to promote increased cutaneous cooling of the skin and a wider area of the energy cone. For these same animals, the FUS beam was repositioned after each sonication to pass over a different section of skin each time to reduce exposure on the same area.

Following ablations, thoracentesis was performed, and saline was removed from the pleural space of the collapsed lung, similar to the drainage of a clinical pleural effusion. After this, animals were allowed to fully recover and treated with antibiotics for a week, with the exception of pigs #1, #10, and #11 which were euthanized (Euthasol (1 mL/4.5 kg)) on the same day as treatment to confirm the presence of ablations at the target areas through gross histology.

One week after treatment, all pigs (except for pigs #1, #10, and #11) underwent a detailed MRI study, using a 1.5 T MRI clinical scanner (Avanto, Siemens Healthcare, Malvern, PA, USA) to assess the quality of ablation. Pigs were then euthanized and examined through gross histology to correlate the results of the MRI.

Pigs #1, #10, and #11 received the same detailed MRI study immediately after the MRgFUS treatment and before being euthanized.

### 2.2. Histology

For all pigs, following euthanization, the skin was inspected for any superficial burns or damage. An incision was then cut parallel to the sternum to expose the external surface of the ribs, which were examined for any subcutaneous damage. The ribs were then cut on the anterior line, and the lungs and heart were removed in one piece and examined for MRgFUS ablations, any signs of additional damage, or abnormalities. On both lungs, longitudinal slices were made down the length of the lung to examine the depth of the ablations and to look for any additional damage. For pigs #5–11, sample biopsies were taken from the ablation area and from healthy non-ablated lung tissue and examined using histopathology.

## 3. Results

In pig #1, the power escalation procedure was carried out with no adverse effects. The pig was observed through MRI during and after the escalation study and showed that ablations up to 300 Watts for 15 s were appropriate for consistent ablation. On all power levels, there were no visible burn marks on the skin, and no damage was found on the surface of the heart or the pericardium. In regards to gross histology, there were lesions in the left (treated) lung that corresponded to MRgFUS ablation locations. As pig #1 was immediately euthanized, there were no detailed longitudinal post-treatment health data, but the pig was stable throughout the entire treatment procedure. This suggested that MRgFUS treatment at these powers and durations can be carried out without any immediate complications.

**Figure 2:**
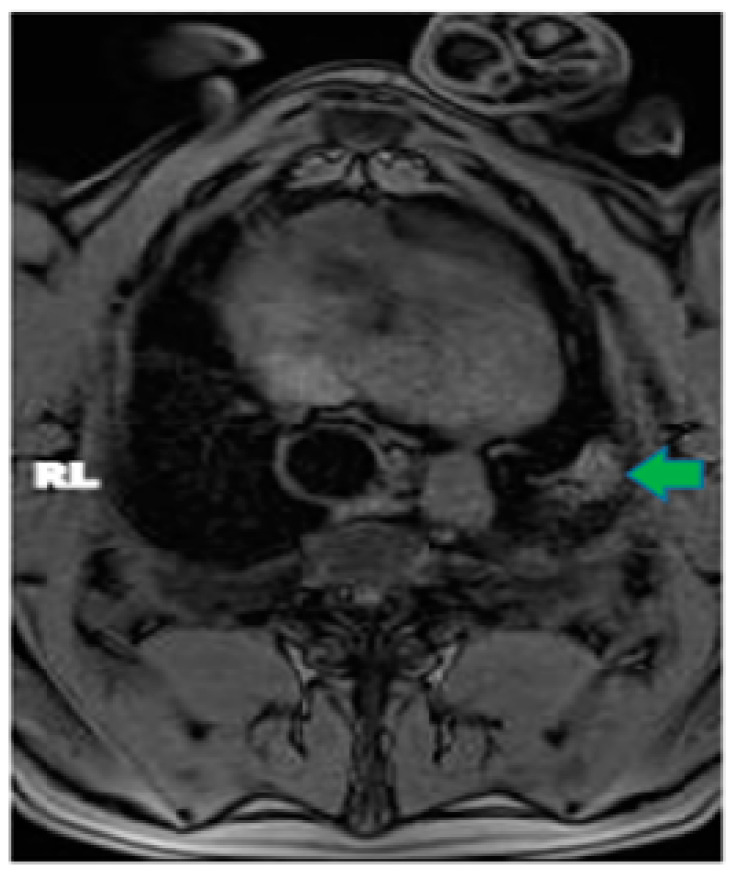
Axial MRI view of pig #8, one-week post MRgFUS ablation treatment. Green arrow points to ablated area in left lung.

**Figure 3:**
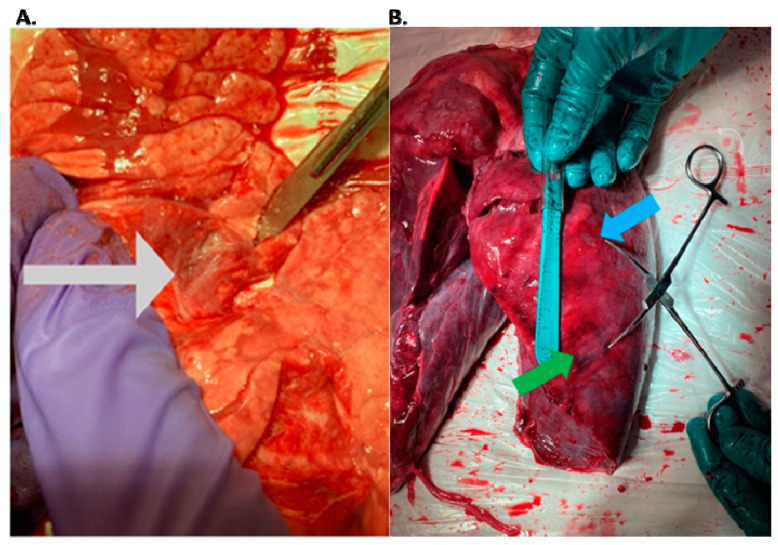
(**A**) Gross histology of the left lung in pig #2. MRgFUS pulmonary lesions are marked with a grey arrow. These lesions demonstrate the depth of the ablations and correspond with hyper intense areas found on follow-up MRI. (**B**) Lung parenchyma of pig #7 following gross histology. Blue arrow shows a 0.8 cm × 0.45 cm ablation in the upper part of the left lung. Green arrow shows a 1.3 cm × 1.1 cm ablation in the lower lobe of the left lung.

In pig #2, the aim was to study the health effects following MRgFUS with multiple (six) doses of a higher power delivered at six different locations but using only two intercostal/skin areas as entry points. Following the six treatments with MRgFUS at 350 Watts for 20 s each, mild burn marks were visible on the skin at the FUS beam entry points. The pig recovered successfully from the anesthesia. Mild wheezing was noticeable in the week-long post-treatment follow-up. After one week, the pig was scanned using MRI and had hyper-intense regions in the left lung that corresponded with the treatment locations (an example is shown Figure 2). The pig was then euthanized and examined utilizing gross histology. At the locations of the MRgFUS treatment, there were dense fibrotic tissues with white discoloration, indicating successful lung parenchyma ablation (Figure 3A). There was no damage observed on the pericardium or heart.

In pigs #3–5, ablations were performed successfully in three spots. Hyper-intensity on the T1w MRI post-treatment correlated with the MRgFUS treatment locations. However, pig #3 had mild cutaneous skin burns and pig #4 showed damage localized in the tissue around the ribs, indicating the ribs may have absorbed some of the ultrasound energy. For pig #5 and the pigs afterwards, we carefully angulated the FUS beams using the pre-treatment MRI to avoid the ribs as much as possible. All the pigs remained healthy with no noticeable health complications in the week-long post-treatment follow-up. Pig #5 was noted to have a small pneumothorax that was present in the left side of the thoracic cavity, which was detected on the one-week post-treatment MRI but had no visible symptoms.

In pigs #6–9, the ablations were reduced to two locations, and several techniques were introduced to reduce collateral damage. The gel pad between the table and the pigs was reduced to 25 mm, and the MRgFUS beam was angulated over a different patch of skin during each ablation. As a result, no further skin burns or tissue damage around the ribs were found on any of the six subsequent pigs.

**Figure 4:**
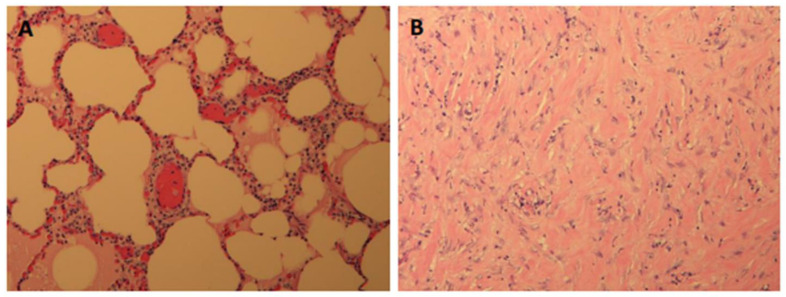
(**A**) Section of normal lung tissue, removed from the middle lobe of the right lung, where no FUS ablation occurred (control); magnification 10×. (**B**) Section of ablated lung tissue, removed from left lung at the FUS ablation target site; magnification 10×. There was an absence of normal alveolar structures and red blood cells and presence of a dense collagen/fibrotic type of structure instead. Both images are from pig #7.

Furthermore, all of the subsequent pigs showed successful ablations in the one-week post-treatment MRI and in their gross histology, with the tissue corresponding to the targeted areas having a hard and fibrotic consistency upon necropsy (Figure 3B). The lung tissue biopsies of the healthy and targeted tissue were examined using histology and confirmed that the FUS-targeted tissues presented a denser fibrotic consistency instead of normal alveolar structures and red blood cells (Figure 4). Pig #9 was unique in that there was a wedge-shaped lesion in the upper right lung. This lesion may have been due to reflection of the MRgFUS beam off the opposing ribcage, as the largest section of the wedge was closest to the ribcage. All of the pigs remained healthy during the post-treatment follow-up week, with no noticeable health complications.

**Figure 5:**
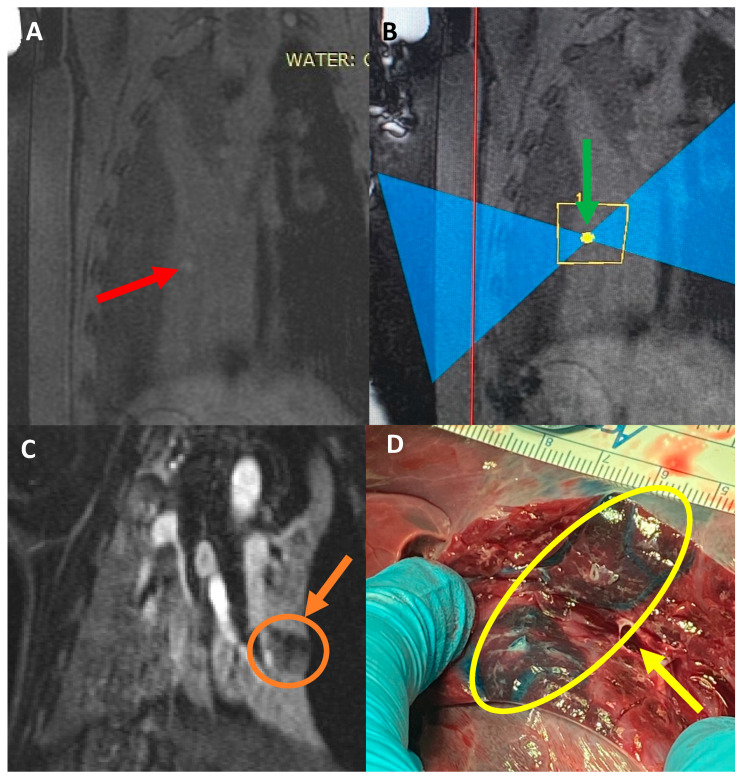
(**A**) Coronal image of pig #11 pre-treatment. Red arrow points toward the location that the Vitamin E mixture was injected into, which shows a hyperintense region. (**B**) Sagittal image during treatment. Green arrow points toward the targeted location. (**C**) Coronal T1 weighted MR image, post-contrast showing the hypo-perfused ablated area. Orange arrow depicts the size of the ablation. (**D**) Necropsy of the targeted area. Yellow arrow depicts the target area generated with the dye mixture. The ablated tissue was firm and directly in the targeted area.

Pigs #10–11 received a liquid mixture of Vitamin E, Evans blue, and food dye in the collapsed lung parenchyma. As planned, the Vitamin E provided a hyper-intense target in the pre-treatment lung MR images (Figure 5A), and each pig received three ablations at 350 Watts for 20 s, each targeting this specific spot (Figure 5B). The ablations were visible on the post-treatment MRI with and without gadolinium contrast, but the absence of perfusion in the ablated areas was noted in the post-gadolinium MR images (Figure 5C), showing the damage of the FUS on the targeted area of the collapsed lung. Upon necropsy, there was no collateral damage noted on the skin, subcutaneous tissue, or muscle. The target sites were highly visible as a blue stain, with an area of ablation in the center, which appeared as a firm, coned area (Figure 5D).

## 4. Discussion

The results of this study demonstrate the transcutaneous in vivo MRgFUS ablation of lung parenchyma behind the rib space, following a controlled hydrothorax combined with intentional lung collapse. Although other studies have demonstrated that flooding the lung parenchyma can conduct FUS beams, we demonstrate that this can be achieved by flooding the pleural space instead. Focal lesions found on the gross histology and MRI tests corresponded with the locations treated with MRgFUS ablation, indicating MRgFUS can accurately target regions of lung parenchyma through the acoustic window created by the hydrothorax. As demonstrated with pigs #10 and #11, we were able to create a visible target location on the MRI and successfully ablate it with MRgFUS. In addition, this method was safe and viable as the pigs demonstrated no collateral damage and no major complications during the post-treatment one-week follow-up phase. While the procedure’s long-term health effects remain unknown, most pigs were noted to have no visible neurological, motor, or breathing problems. These preliminary results indicate that MRgFUS combined with a hydrothorax and collapsed lung has the potential to be a feasible treatment technique for the ablation of lung tissue.

This procedure is encouraging as MRgFUS combined with a hydrothorax and lung collapse has the potential to have fewer side effects than traditional surgical treatment and FUS OLF. OLF requires the creation of intentional pulmonary edema, thus carrying a high risk of infection and multiple other potential complications. The procedure described here only requires the flooding of the pleural space outside of the lung, with a decreased risk of complications when compared to that of OLF, since fluid can reliably be removed from the pleural space following treatment. In addition, as this procedure requires fewer or no incisions when compared to traditional surgical techniques, it is more suitable for patients who are high-risk surgical candidates and can reduce the risk of post-surgical infection and other complications.

At the beginning of this study, we had a very detailed concept of all the necessary steps for an effective study, but there was not a lot of background knowledge for some of the fundamental details that had to be established. For example, there was no known baseline power for MRgFUS treatment of lung tissue through the hydrothorax, and it was unknown which power level would deliver consistent results with minimal side effects. To discover the power required, we did a progressive escalation study, with the first pig receiving eight transcutaneous ablations, the second animal receiving six, etc., until we found an appropriate power of approximately 350 Watts for 20 s, which is the amount of time that a safe breath-hold could be performed while mechanically ventilating the anesthetized pig. However, as this technique was being piloted, there were mild superficial cutaneous burns found on the first two pigs treated. We were able to remedy the issue of skin burns by utilizing a thinner 25 mm gel pad between the skin and the water-cooled table surface, promoting the more efficient cooling of the skin. Another technique utilized was angulating the beam to target a different section of skin in the ablation to reduce the amount of superficial exposure. These issues demonstrated that gel layer thickness and beam angulation should be considered for future experiments.

One limitation for this entire study was the transducer that was used for the MRgFUS treatment. For this project, an Exablate 2000OR Body system was used with a sectored concentric-ring phased array transducer, mainly designed for thermal ablation treatment of abdominal soft tissue, like uterine fibroids. Despite having some electronic steering capabilities as well as a focal depth range of 60–220 mm [16], which can cover the entire hemisphere of an adult’s chest cavity, this transducer has a diameter of 120 mm, making it a suboptimal design for shooting between the ribs for lung tissue ablation. The results of this pilot study were good but a better transducer design and different array configurations could improve the efficacy and accuracy of this technique even further. If this technique is adopted for human use, more accurate transducers that can ablate between the ribs will need to be developed. These transducers currently do not exist and will need to be created and tested.

Further limitations include the size of the tissue volume that can be ablated in one shot by the Exablate2000 system. The system is capable of ablating tissue up to 12 mm × 12 mm × 30 mm reliably, but tumors that are larger than this size may need a multiple-shoot volumetric approach, like the Federal-Drug-Administration-approved technique for the treatment of uterine fibroids. This has also been studied in prostate cancer and benign thyroid nodules [17,18], but studies regarding multiple HIFU treatments for lung cancer have not been conducted.

Pigs #10 and #11 demonstrated that we can create an artificial target visible on MR images and during histopathology, successfully target it, and ablate it. While the amount of Vitamin E was low on pig #10, and the target location was very faint, making it difficult to target the specific region, we rectified this and increased the mixture volume for the following pig (pig #11), thus making the target region more visible on the MR images, as well as during the necropsy studies. Future experiments can utilize higher amounts of Vitamin E, perhaps in conjunction with Matrigel, to create a more localized target for ablation that is even more visible on MRI.

Overall, MRgFUS combined with a controlled hydrothorax and lung collapse allowed us to accurately target the deep tissue of lung parenchyma without the need to flood the lung internally with saline or another fluid. All the subjects remained healthy after the treatment and at a week-long post-treatment follow-up.

Following refinement, there were no skin burns from MRgFUS and we were able to ablate an artificially generated target location. Future experiments can use the baselines established in this study to fine-tune this technique and to improve our methodologies and equipment.

## 5. Conclusions

This experiment was a demonstration of a novel technique for MRgFUS that uses controlled lung collapse and a hydrothorax to create an acoustic window to accurately target the ablation of deep lung parenchyma. We were able to ablate specific regions of the lung in a porcine model, thus creating a new treatment methodology that may be used to treat benign or malignant lung masses. MRgFUS has the potential to offer an alternative to surgical resection and ablative stereotactic body radiation therapy [19] and to increase lung cancer survival rates while decreasing morbidity, as it is less invasive than traditional surgery and may be used for the treatment of patients who are not stable enough to undergo a surgical intervention.

## Figures and Tables

**Table 1:** Number of lung ablation targets, power, and duration of ablations.

Pig #	Breed	Weight	Number of Ablation Targets	Power and Duration of Ablations	
1	Mini Yucatan	49 kg	8	(1) 45 Watts—15 s	(5) 150 Watts—30 s
				(2) 75 Watts—30 s	(6) 150 Watts—60 s
				(3) 105 Watts—30 s	(7) 150 Watts—60 s
				(4) 105 Watts—60 s	(8) 300 Watts—15 s
2	Mini Yucatan	31 kg	6	350 Watts—20 s (@ each location)	
3	Yorkshire	53 kg	3	2× 350 Watts—20 s (@ each location)	
4	Yorkshire	53 kg	3	350 Watts—20 s (@ each location)	
5	Yorkshire	53 kg	3	300 Watts—20 s (@ each location)	
6	Yorkshire	60 kg	2	350 Watts—20 s (@ each location)	
7	Yorkshire	60 kg	2	350 Watts—20 s (@ each location)	
8	Yorkshire	63 kg	2	350 Watts—20 s (@ each location)	
9	Yorkshire	53 kg	2	350 Watts—20 s (@ each location)	
10	Yorkshire	50 kg	1	3× 350 Watts—20 s	
11	Yorkshire	50 kg	1	3× 350 Watts—20 s	

## Data Availability

Data are contained within the article.
